# Catching the fish with the worm: a case study on eDNA detection of the monogenean parasite *Gyrodactylus salaris* and two of its hosts, Atlantic salmon (*Salmo salar*) and rainbow trout (*Oncorhynchus mykiss*)

**DOI:** 10.1186/s13071-018-2916-3

**Published:** 2018-06-04

**Authors:** Johannes C. Rusch, Haakon Hansen, David A. Strand, Turhan Markussen, Sigurd Hytterød, Trude Vrålstad

**Affiliations:** 1 Norwegian Veterinary Institute, P.O. Box 750, Sentrum, NO-0106 Oslo, Norway; 2 Department of Biosciences, University of Oslo, P.O. Box 1066, Blindern, NO-0316 Oslo, Norway; 3 Faculty of Veterinary Medicine, Norwegian University of Life Sciences, P.O. Box 8146, Dep, NO-0033 Oslo, Norway

**Keywords:** Environmental DNA, Multiplex PCR, Droplet digital PCR (ddPCR), Internal transcribed spacer (ITS), Mitochondrial DNA (mtDNA), Invasive species

## Abstract

**Background:**

Environmental DNA (eDNA) monitoring is growing increasingly popular in aquatic systems as a valuable complementary method to conventional monitoring. However, such tools have not yet been extensively applied for metazoan fish parasite monitoring. The fish ectoparasite *Gyrodactylus salaris*, introduced into Norway in 1975, has caused severe damage to Atlantic salmon populations and fisheries. Successful eradication of the parasite has been carried out in several river systems in Norway, and Atlantic salmon remain infected in only seven rivers, including three in the Drammen region. In this particular infection region, a prerequisite for treatment is to establish whether *G. salaris* is also present on rainbow trout upstream of the salmon migration barrier. Here, we developed and tested eDNA approaches to complement conventional surveillance methods.

**Methods:**

Water samples (2 × 5 l) were filtered on-site through glass fibre filters from nine locations in the Drammen watercourse, and DNA was extracted with a CTAB protocol. We developed a qPCR assay for *G. salaris* targeting the nuclear ribosomal ITS1 region, and we implemented published assays targeting the mitochondrial cytochrome-b and NADH-regions for Atlantic salmon and rainbow trout, respectively. All assays were transferred successfully to droplet digital PCR (ddPCR).

**Results:**

All qPCR/ddPCR assays performed well both on tissue samples and on field samples, demonstrating the applicability of eDNA detection for *G. salaris*, rainbow trout and Atlantic salmon in natural water systems. With ddPCR we eliminated a low cross-amplification of *Gyrodactylus derjavinoides* observed using qPCR, thus increasing specificity and sensitivity substantially. Duplex ddPCR for *G. salaris* and Atlantic salmon was successfully implemented and can be used as a method in future surveillance programs. The presence of *G. salaris* eDNA in the infected River Lierelva was documented, while not elsewhere. Rainbow trout eDNA was only detected at localities where the positives could be attributed to eDNA release from upstream land-based rainbow trout farms. Electrofishing supported the absence of rainbow trout in all of the localities.

**Conclusions:**

We provide a reliable field and laboratory protocol for eDNA detection of *G. salaris*, Atlantic salmon and rainbow trout, that can complement conventional surveillance programs and substantially reduce the sacrifice of live fish. We also show that ddPCR outperforms qPCR with respect to the specific detection of *G. salaris*.

## Background

*Gyrodactylus salaris* Malmberg, 1957 (Monogenea) is an ectoparasite first described on the skin of Atlantic salmon *Salmo salar* (L. 1758), where it attaches itself to the host with a haptor, a specialized attachment organ consisting of a large disc with 16 peripheral articulated marginal hooks and a single pair of ventrally orientated hamuli [1]. This ~500 μm long parasite [2] has also been found on other salmonids such as rainbow trout *Oncorhynchus mykiss* (Walbaum, 1792) [3], brown trout *Salmo trutta* (L., 1758) and Arctic charr *Salvelinus alpinus* (Linnaeus, 1758) [4]. While most species and populations of fish which act as hosts, including Baltic populations of Atlantic salmon, do not experience serious consequences of a *G. salaris* infection [1, 5], Atlantic populations of salmon are highly susceptible to *G. salaris* resulting in high mortality rates in mainly Norwegian populations (see below). Rainbow trout is less susceptible, and can sustain infections for long periods, often at low intensities making it an important host when considering spreading between fish farms in Europe [6].

In 1975, *G. salaris* was detected in Norway for the first time [7–9]. The parasite has since caused severe damage to several Atlantic salmon populations [1]. Altogether, fish in 50 rivers in Norway have been infected by *G. salaris* and extensive eradication programs, mostly using pesticides such as rotenone, have been carried out in several of these watercourses [10] since 1981 [11]. Over the last 15 years [12], the eradication programs have been highly successful and to date the parasite is present only in seven rivers [10]. To document the absence of *G. salaris* in Norwegian river systems and to detect new infections at an early stage, large-scale national surveillance programs are carried out every year [10, 13]. Present surveillance is based on the catching and killing of numerous Atlantic salmon juveniles in rivers and farms, as well as rainbow trout reared in farms, for morphological screening for the presence or absence of *G. salaris*. In 2016 alone, 6981 fish were killed and examined [10, 13].

One of the remaining regions where *G. salaris* is still present is the Drammen region (Buskerud and Vestfold County) in southern Norway, consisting of the rivers Drammenselva, Lierelva and Sandeelva (hereafter referred to by their Norwegian names). The infection region including a control area is described in the Norwegian legislation [14]. A strategy to implement treatment of this region has not yet been conclusively devised by the Norwegian authorities, as this watercourse in many aspects is more complicated than previously treated systems. This results from three basic factors. First, rainbow trout in the system upstream of the current migration barriers for salmon have a history of infection with *G. salaris* [8]. Secondly, Drammenselva contains a much higher fish species diversity than other treated rivers, which mainly contain salmonids. Thirdly, this river discharges into a large estuary with surface water containing low salinity (< 2%) where *G. salaris* can survive for longer periods [15]. In order to decide on measures regarding treatment of this water system, exact knowledge of the status of infections with *G. salaris* in the area is a prerequisite. Rainbow trout farms in the northern parts of the Drammen watercourse were infected with *G. salaris* in the mid-1980s and later there have been both documented [16, 17] and anecdotal reports of free-living rainbow trout in the system. There is thus a possibility that free-living rainbow trout are still present in the system and these might have sustained the introduced *G. salaris* infection from the 1980s. Therefore, a surveillance program [18, 19] has been established to detect any possible presence of *G. salaris* on free-living populations of rainbow trout upstream of the anadromous parts of the Drammenselva catchment.

Standard surveillance for fish parasites, including the surveillance programs for *G. salaris* in Norway, involves capture and euthanasia of fish, prior to manual examination for the presence of parasites. This is both costly and labour-intensive, and results in the sacrifice of a large number of usually infection-free healthy fish. In recent years, capturing, amplifying and detecting species-specific DNA fragments of several species in water samples has been established as an accurate low-cost alternative or a complement to traditional monitoring [20–23]. This approach, harnessing so-called environmental DNA (eDNA), makes use of the knowledge that all organisms shed cells into their surroundings (excretion, mucus layers, abrasions of epithelial tissue, gametes) [24, 25]. For eDNA monitoring of natural waters, the eDNA content represents to a large extent a snap-shot of the present living species, with a time lag of only some weeks after a species has disappeared from the system until eDNA is no longer detectable [26]. Results are delivered relatively fast and efficiently [27], often at lower agent-prevalence than through traditional methods [28].

To complement conventional surveillance methods for *G. salaris*, we aimed at developing an eDNA approach for targeted detection of the parasite-host combination in water samples. We applied this method in a case-study, where eDNA detection by means of species specific quantitative PCR (qPCR) and droplet digital PCR (ddPCR) was used as a supplement to standard surveillance methods for *G. salaris*, Atlantic salmon and rainbow trout in the Drammen infection region, Norway.

## Methods

### Description of the study area

**Fig. 1:**
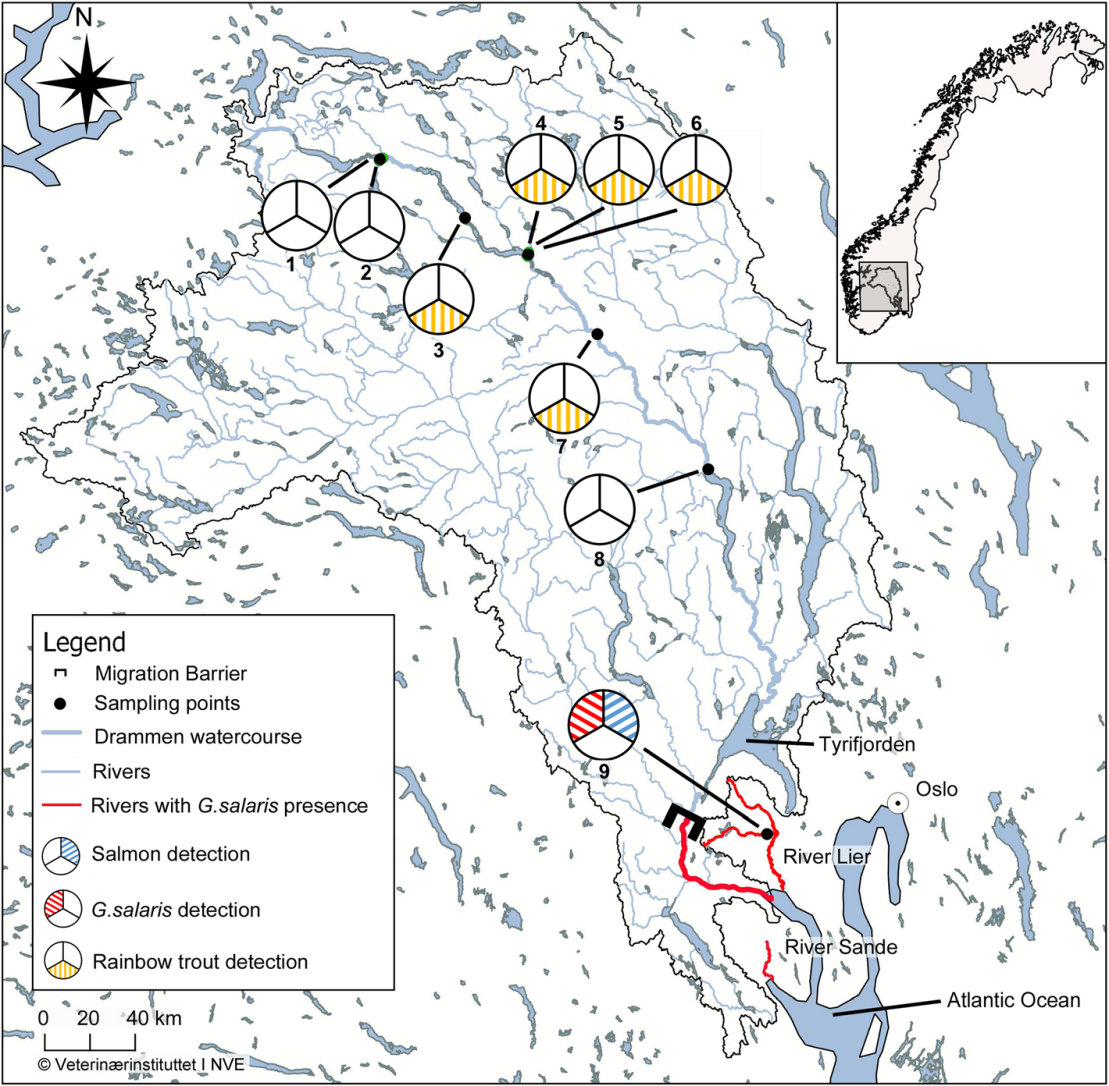
Map of the Drammen watercourse region with all sampling locations and its location within Norway. Green points represent localities sampled. The thick blue line represents the Drammen watersystem, the thin blue lines represent the main rivers, the red lines indicate rivers where *G. salaris* is present and the black lines outline the Drammenselva drainage basin. The numbers refer to the sampling sites in Table 1. Pie charts: blue colour indicates detection of Atlantic salmon, red indicates detection of *Gyrodactylus salaris* and yellow indicates detection of rainbow trout. Rivers flow north to south

One part of this study was conducted in the northern part of the Drammenselva watercourse (Oppland County) where a presence of wild rainbow trout populations is possible and the status of *G. salaris* is unknown. The other part of the study was conducted in Lierelva (Buskerud County), a small river in the Drammen infection region where Atlantic salmonhasbeen infected with *G. salaris* since 1987 [1]. Drammenselva drains from the Jotunheimen Mountains in the north, down to Drammensfjorden (Buskerud and Vestfold Counties) which connects the watercourse with the Atlantic Ocean (Fig. 1). The infection region in Drammen incorporates three of the remaining seven rivers in Norway where *G. salaris* is still present. These are: Drammenselva, Lierelva (both Buskerud County) and Sandeelva (Vestfold County) (Fig. 1), in all of which Atlantic salmon is present. Lierelva and Sandeelva are smaller rivers with catchment sizes of 309.6 and 193.4 km^2^, respectively, while Drammenselva drains from a much larger area (17,110.8 km^2^). In the northern part of the Drammen watercourse (see Fig. 1), several rainbow trout producers can be found. Fish in farms in this area were infected by *G. salaris* in the mid-1980s and there were many reports of escaped fish from the farms [14]. However, the fish populations in the farms were eradicated and all these farms were declared free from *G. salaris* in 1987 [29]. In 1986, *G. salaris* was also diagnosed from farmed rainbow trout and salmon in the Lake Tyrifjorden which is a part of the Drammen watercourse [8, 30]. The fish populations in these farms were also eradicated, but a short time later, the parasite was detected on salmon juveniles from Drammenselva and Lierelva [30].

### Sample locations

The sampling sites included eight localities in the northern part of the Drammenselva watercourse, (Fig. 1, Table 1). These sampling sites were chosen as part of a monitoring program [19] and with the intention of both declaring this region free from *G. salaris* and mapping the presence of free-living rainbow trout. One of these eight sampling sites was a fish pond at a local trout farm that served as a rainbow trout positive field control sample. The ninth sample was chosen as a positive field control sample for only *G. salaris* and Atlantic salmon and collected from a stretch in Lierelva (Fig. 1), a river with a confirmed presence of Atlantic salmon infected with *G. salaris*.

**Table 1:** List of sampling sites including location, sampling date and amount of water filtered

Site no.	Site name	Water filtered (l)	Coordinates	Date
1	Storåne at Ala camping	5 (×2)	61.1473N, 8.7121E	26.06.2017
2	Storåne at Tørpegårdsvegen/bru	5 (×2)	61.1522N, 8.7250E	26.06.2017
3	Trout farm	5 (×2)	61.0379N, 9.0466E	14.11.2016
4	Leireelvi at Leira/Garlivegen	5 (×2)	60.9742N, 9.2936E	26.06.2017
5	Leireelvi at Leira camping	5 (×2)	60.9680N, 9.2884E	26.06.2017
6	Lake Strondafjorden at Faslefoss	5 (×2)	60.9671N, 9.2889E	26.06.2017
7	River Begna at Bagn	5 (×2)	60.8198N, 9.5612E	26.06.2017
8	River Begna at Nes	5 (×2)	60.5628N, 9.9929E	26.06.2017
9	Lierelva at Sjåstad	5 (×4)	59.8580N, 10.2213E	31.08.2017

Within the area where rainbow trout farms can be found, the locations of sample nos. 4 and 5 were chosen based on information obtained from the local authorities prior to the field work. These samples were taken in streams flowing into the main watercourse in order to avoid positive detections due to outlet water from farms situated upstream in the main watercourse. For an indication of the sensitivity of the rainbow trout eDNA assay for detection in the field, three samples (nos. 6, 7 and 8) were taken from the main watercourse at different distances from the rainbow trout farms. Samples 1 and 2 were taken upstream of the area containing rainbow trout farms.

### Electrofishing and *Gyrodactylus* counts

Electrofishing was carried out in rivers and tributaries in the Drammen watercourse to reveal the possible presence of rainbow trout, using this standard surveillance method. The area examined was chosen on site according to local conditions (stream size, depth, water flow). Electrofishing was also conducted in Lierelva to collect salmon juveniles for estimation of the infection prevalence and intensity of *G. salaris* in the same locality as water samples were taken. Fish captured for further examination were euthanised following the strict codes of practice in force in Europe, preserved intact in 96% ethanol and later examined for the presence of *Gyrodactylus* spp. using a stereo microscope (Leica MZ 7.5, Leica microsystems, St. Gallen, Switzerland).

### Water filtering for eDNA sampling

At each sampling location, duplicate water samples of 5 L (2 × 5 l) were collected and filtered on site on to glass fibre filters (47 mm AP25 Millipore, 2 μm pore size, Millipore, Billerica, USA) using a portable peristaltic pump (Masterflex E/S portable sampler, Masterflex, Gelsenkirchen, Germany), tygon tubing (Masterflex) and an in-line filter holder (Millipore) according to Strand et al. [31]. At Lierelva, four samples were taken instead of two as this river was intended as a positive field control for *G. salaris* and Atlantic salmon. Filters were placed in separate 15 ml Falcon tubes containing cetyl trimethyl ammonium bromide (CTAB) buffer and stored on ice directly after filtration. Upon arrival at the laboratory the samples were stored at -20 °C until further analysis. As a safety precaution and part of the filtering protocol, the entire equipment was disinfected with a 10% bleach solution after use at each location. Thus, any residual eDNA was broken down and contamination was prevented. Before further sampling, the tubes were rinsed with sodium thiosulphate to neutralise the bleach solution, and then flushed with ambient river water directly before sampling.

### DNA extraction

DNA was extracted from the filters according to a CTAB protocol described in Strand et al. [31], with the exception that the CTAB buffer contained no added 1% 2-mercapto-ethanol. During extraction each filter was split into two subsamples (A and B) due to volume restrictions imposed by centrifuge size and extracted separately. An environmental control and a blank extraction control were included as a precaution to detect any possible contamination during DNA extraction. The blank extraction control consisted of a Falcon tube containing the CTAB buffer but no filter, which was then processed in the same way as all other tubes containing buffer and filters. The environmental control used in this study consisted of an Eppendorf tube containing 200 μl PCR-grade water. This tube remained open in the fume hood throughout the entire process of extraction.

### PCR-based assays for eDNA detection of *G. salaris*, rainbow trout and Atlantic salmon

A quantitative PCR assay (qPCR) using species-specific primers and a minor groove binder (MGB) probe targeting the *G. salaris* internal transcribed spacer region 1 (ITS1) was developed. The ITS1 sequence published as GenBank accession no. DQ898302 was used as template and the specificity of the designed primers and probe was checked against closely related species and other species that might be present in Norwegian watercourses: *G. salmonis* Yin & Sproston, 1948 (GQ368233), *G. truttae* Gläser, 1974 (AJ132260), *G. lucii* Kulakovskaya, 1952 (EU304811), *G. arcuatus* Bychowsky, 1933 (JN703797) and *G. derjavinoides* Malmberg, Collins, Cunningham & Jalali, 2007 (EU304810). Multiple sequences were aligned using AlignX (Vector NTI Advance 11.5, Invitrogen, Carlsbad, USA). The design of primers and probe was performed manually, targeting ITS1 sequence regions displaying the highest sequence diversity between *G. salaris* and the species listed above. The final primer and probe sequences (Table 2) partly overlap with those previously published for this parasite [32] and their specificity was confirmed through matching them against the database of the National Centre for Biotechnology Information (NCBI, http://www.ncbi.nlm.nih.gov/) nucleotide database using the Basic Local Alignment Search Tool (BLASTn). The aim of the new qPCR assay was to attempt to obtain the best possible sensitivity and specificity for eDNA applications. Similar to the assay Collins et al. [32] designed, the newly designed assay is not able to distinguish between *G. salaris* and *G. thymalli *Žitňan, 1960 as these two species have indistinguishable ITS sequences [33].

**Table 2:** Primers and probes for *Gyrodactylus salaris*, rainbow trout (*Oncorhynchus mykiss*) and Atlantic salmon (*Salmo salar*) used in the present study. The probes used are TaqMan MGB probes with either Fam or Hex reporter dyes

Target species/gene	Name	Primer/probe	Sequence (5'-3')	Reference
*G. salaris*/ITS	G.sal208F	Forward	GGTGGTGGCGCACCTATTC	Present study
	G.sal149R	Reverse	ACGATCGTCACTCGGAATCGAT	Present study
	G.sal188P	Probe	(FAM)CAAGCAGAACTGGTTAAT(MGBNFQ)	Present study
*G. salaris*/ITS	F	Forward	CGATCGTCACTCGGAATCG	Collins et al. [32]
	R	Reverse	GGTGGCGCACCTATTCTACA	Collins et al.[32]
	Gsal2	Probe	(FAM)TCTTATTAACCAGTTCTGC(MGBNFQ)	Collins et al. [32]
*O. mykiss*/*cytb*	RBTF	Forward	AGTCTCTCCCTGTATATCGTC	Wilcox et al. [35]
	RBTR	Reverse	GATTTAGTTCATGAAGTTGCGTGAGTA	Wilcox et al. [35]
	RBTP	Probe	(FAM)CCAACAACTCTTTAACCATC(MGBNFQ)	Wilcox et al. [35]
*S. salar*/*cytb*	Salmonid Cyt B FOR	Forward	CGGAGCATCTTTCTTCTTTATCTGT	Matejusova et al. [34]
	S. salar REV	Reverse	ACTCCGATATTTCAGGTTTCTTTATATAGA	Matejusova et al. [34]
	S. salar Cyt B Probe	Probe	(HEX)CCAACAACTCTTTAACCATC-(MGBNFQ)	Matejusova et al. [34]

The assays used for eDNA-detection of Atlantic salmon and rainbow trout (Table 2) follow Matejusova et al*.* [34] and Wilcox et al*.* [35], respectively. These were successfully tested on DNA extracts from tissue of Atlantic salmon and rainbow trout before use in the current study (data not shown). The ddPCR assay for *G. salaris*, rainbow trout and Atlantic salmon applied the same primers and probes as the qPCR.

### Evaluation of qPCR and ddPCR assay specificity

The specificity of the assay was tested on DNA extracts of *G. salaris* collected from three different locations in Norway in addition to DNA extracts from the following other species present in the collection at the NVI: *G. thymalli*, *G. salmonis*, *G. arcuatus*, *G. lucii* and *G. derjavinoides*. Species identification of these samples had been done previously by sequencing of ITS (results not shown). We also ran the same samples with the previously published assay [32] to compare the specificity and sensitivity of the assays. ddPCR applies the same primers and probes as qPCR and the specificity was tested on *G. derjavinoides* due to the low level of cross amplification shown in a previously published assay [32]. The ddPCR assay was also tested on isolates of *G. salaris* obtained from fish from Lierelva to determine optimal annealing temperature.

### qPCR and ddPCR protocols for *G. salaris* eDNA detection

All qPCR analyses were carried out on an Mx3005P qPCR system (Stratagene, San Diego, USA). Droplet digital PCR was performed on a QX200 AutoDG Droplet Digital PCR System (Bio-Rad, Hercules, USA).

For qPCR detection of *G. salaris*, three qPCR replicates were run for each eDNA extract in the following 25 μl reactions: 1.25 μl of PCR-grade water, 12.5 μl of ExTaq mastermix (Takara Biotechnology, Dalian, China), 1.5 μl of each 10 μM primer (forward and reverse), 0.75 μl of 10 μM probe, 0.5 μl of Rox II reference dye and 5 μl of eDNA template. The qPCR cycling conditions were as follows: an initial denaturation at 95 °C for 15 min; 45 cycles of denaturation at 94 °C for 30 s, annealing at 54 °C for 45 s, and extension at 72 °C for 1 min; followed by a final elongation phase at 72 °C for 10 min.

The following 22 μl reactions were run for each eDNA extract on ddPCR: 11 μl ddPCR Supermix for probes - no dUTP (Bio-Rad), 1.98 μl of each 10 μM primer, 0.55 μl of 10 μM probe, 0.49 μl PCR-grade water and 1 μl of restriction-enzyme mix consisting of 0.2 μl HindIII, 0.1 μl buffer (10×) and 0.7 μl PCR-grade water and 5 μl of DNA sample. The optimal primer-probe concentration was determined to be 900:250 and the optimal annealing temperature of 58 °C was confirmed through amplification tests along a temperature gradient. Here, we used the *Hind*III restriction enzyme to fragment the repetitive multi-copy ITS regions within the nuclear ribosomal DNA in order to ensure that the targeted DNA copies were distributed among different droplets for accurate quantification.

To allow for sufficient time for the restriction enzymes to digest, the plate was sealed using Microseal ‘B’ plate sealing film (Bio-Rad), wrapped in tin foil and left on the bench for 20 min. Droplet generation in the QX200 AutoDG Droplet Digital PCR System (Bio-Rad) creates an emulsion with 20 μl of the 22 μl originally pipetted into each well, resulting in a 10% loss of template and mastermix. After generation of the droplets, the new plate was immediately transferred to a TM100 thermocycler (Bio-Rad) and the QX200 Droplet Digital PCR system (Bio-Rad) with the following cycling conditions: An initial denaturation at 95 °C for 10 min; 45 cycles of denaturation at 94 °C for 30 s, annealing at 58 °C for 60 s; followed by a final elongation phase at 58 °C for 10 min. The threshold for a positive sample was set at three positive droplets per well according to Dobnik et al*.* [36]. To ensure the validity of each run, positive and blank PCR controls containing *G. salaris* DNA and distilled water, respectively, were run on each plate for both qPCR and ddPCR.

To be able to detect *G. salaris* and Atlantic salmon simultaneously in future surveillance programmes in Norwegian rivers, we also tested a duplex method using the same primers and probes as for the singleplex reactions. This duplex method was set up by running the following 22 μl reactions for each eDNA extract in duplicates: 11 μl ddPCR Supermix for probes - no dUTP (Bio-Rad), 0.99 μl of 20 μM of Salmonid Cyt B FOR and S. salar REV primers, 0.55 μl of 10 μM S. salar Cyt B Probe, 0.99 μl of 20 μM of G.sal208F and G.sal149R primers, 0.275 μl of 20 μM G.sal188P probe, 0.215 μl PCR-grade water and 1 μl of restriction-enzyme mix consisting of 0.2 μl *Hind*III, 0.1 μl buffer (10×), 0.7 μl PCR-grade water and 5 μl of DNA sample. The optimal primer-probe concentration for both assays had been determined to be 900:250. The same cycling conditions were used as in the *G. salaris* singleplex reaction.

For qPCR detection of *O. mykiss*, three qPCR replicates were run for each eDNA extract in the following 12 μl reactions: 2.35 μl of PCR-grade water, 6.25 μl of ExTaq mastermix (Takara), 0.3 μl of 10 μM RBTF forward primer and 0.6 μl of 10 μM RBTR reverse primer, 0.25 μl of 10 μM RBTP probe, 0.25 μl of Rox II reference dye and 2 μl of DNA template. The qPCR (Stratagene) cycling conditions were as follows: an initial denaturation at 95 °C for 1 min; 45 cycles of denaturation at 94 °C for 30 s, annealing at 54 °C for 45 s and extension at 72 °C for 1 min; followed by a final elongation phase at 72 °C for 10 min. We used a cut-off at Cq 41 for the rainbow trout-assay, similar to the suggestion for eDNA qPCR detection cut-off in Agersnap et al*.* [37].

For the singleplex ddPCR detection of rainbow trout, the following 22 μl reactions for each eDNA extract were run in duplicates: 11 μl ddPCR Supermix for Probes - no dUTP (Bio-Rad), 0.99 μl of RBTF 10 μM forward primer, 1.98 μl of 10 μM RBTR reverse primer, 0.55 μl of 10 μM RBTP probe, 2.48 μl PCR-grade water and 5 μl of DNA template. The optimal primer-probe concentration for both assays had been determined to be 450:900:250 for forward primer, reverse primer and probe, respectively, which follows the suggestions in Wilcox et al*.* [35]. The same cycling conditions were used as in all other ddPCR reactions.

### Calculation of eDNA copies

The number of eDNA copies (for each target species) per litre of water for each sample is calculated according to the following formula, also used by Agersnap et al*.* [37]:

$$ {C}_L=\frac{C_{rdd}\ast \left(\frac{V_e}{V_r}\right)}{V_w} $$

where C_L_ is the number of target-eDNA copies per litre of filtered water, C_rdd_ is the ddPCR calculation of eDNA copy numbers per reaction volume (20 μl), adjusted for a 10% loss during droplet generation, V_e_ is the total elution volume after extraction, V_r_ is the volume of eluted extract used in the ddPCR reaction, V_w_ is the volume of filtered water. The copy numbers of both subsamples (A and B) were added together, thus revealing the number of eDNA copies per litre of any given sample. Calculation of eDNA copy numbers per reaction volume was performed by the QuantaSoft software (v.1.7.4, Bio-Rad) and was estimated using the ratio between positive and negative droplets within a sample, using Poisson statistics.

## Results

### qPCR assay optimisation and specificity tests

The current assay proved slightly more sensitive (by ~0.5 Cq) towards *G. salaris* than the assay in Collins et al*.* [31]. The PCR efficiency ([E = ^10-1/slope^] -1) × 100 calculated from triplicates of non-diluted and three 10-fold dilutions of a DNA extract originating from a single parasite, was 100 % (Cq = 20.5 to 30.6, slope = 3.312) (not shown). The qPCR assay for *G. salaris* yielded negative qPCR results for all other species except *G. salaris* (and *G. thymalli* as previously explained), except for a low level of cross-reaction towards the tested specimen of *G. derjavinoides* (Cq = 35.6).

### Optimisation of ddPCR assay and specificity tests

Both the qPCR assay (primers and probes) for *G. salaris* developed in this study and the assays for rainbow trout and Atlantic salmon [32, 34] were transferable to the ddPCR platform without further optimisation, using an annealing temperature of 58 °C. Unlike the qPCR assay however, the ddPCR assay showed no signs of cross amplification of *G. derjavinoides*.

### eDNA monitoring of *G. salaris*, Atlantic salmon and rainbow trout

The positive control field samples for *G. salaris* taken from Lierelva all yielded positive results in qPCR with Cq-values ranging from 24.76 to 35.86, and in ddPCR with eDNA copies/l ranging from 371,440 to 560, respectively. For Atlantic salmon, the eDNA copy numbers ranged from 10,160 (sample 9/2) to 7520 (sample 9/4) (Table 3) at an average of 8948 copies (± SD = 945).

**Table 3:** Overview of results from qPCR and ddPCR analyses for *Gyrodactylus salaris* (ITS), *Oncorhynchus mykiss* (CytB) and *Salmo salar* (CytB) at each sampling site. List of sampling sites including amount of water filtered, number of samples per site (each sample constitutes of one filter), the Cq value (from qPCR) and number of eDNA copies per litre (ddPCR) from all filters taken at each point, respectively. eDNA copies per litre are abbreviated as eDNA/l. No detection is indicated with a minus (-) for qPCR and a zero for ddPCR and those samples where analysis was not applicable are indicated with NT

Site no.	Site name	Sample	Volume (l)	*Gyrodactylus salaris*		*Oncorhynchus mykiss*		*Salmo salar*	
				qPCR^a^	ddPCR^b^	qPCR^a^	ddPCR^a^	qPCR	ddPCR^b, a^
1	Storåne at Ala camping	1	1	-	-	-	0	-	0
		2	1	-	-	-	0	-	0
2	Storåne at Tørpegårdsvegen/bru	1	1	-	-	-	0	-	0
		2	1	-	-	-	0	-	0
3	Trout farm	1	1	-	-	17.48	7,848,000	-	0
		2	1	-	-	17.50	8,800,000	-	0
4	Leireelvi at Leira/Garlivegen	1	1	-	-	29.62	1624	-	0
		2	1	-	-	29.09	3816	-	0
5	Leireelvi at Leira camping	1	1	-	-	30.05	2240	-	0
		2	1	-	-	30.02	2124	-	0
6	Lake Strondafjorden at Faslefoss	1	1	-	-	32.3	560	-	0
		2	1	-	-	31.68	576	-	0
7	River Begna at Bagn	1	1	-	-	> cut-off^c^	0	-	0
		2	1	-	-	36.91	22	-	0
8	River Begna at Nes	1	1	-	-	-	0	-	0
		2	1	-	-	-	0	-	0
9	River Lierelva at Sjåstad	1	1	34.52	560	-	NT	NT	9200
		2	1	33.56	840	-	NT	NT	10,160
		3	1	33.94	864	-	NT	NT	7520
		4	1	24.89	371,440	-	NT	NT	8912

^a^Run as singleplex

^b^Run as duplex

^c^Cut-off value was set at Cq 41

The two positive control field samples for rainbow trout obtained at the trout farm in 2016 tested positive for rainbow trout (Cq 17.48 and Cq 17.50; 8,800,000 eDNA copies/l and 7,848,000 eDNA copies/l, respectively) (see Table 3). Of the other 18 water samples that were collected at the eight sampling points in June and August 2017, five were positive for rainbow trout. Positive samples for rainbow trout were obtained from locations 3, 4, 5, 6 and 7 (see Table 3). One of the five positive sampling sites (no. 6) was at the outlet of the lake into which all the rainbow trout farms drain, while another (no. 7) was found in the main river 25 km downstream of the outlet. According to new information from local authorities we received upon enquiry after our analyses detected rainbow trout DNA in samples 4 and 5, these locations were indeed also situated roughly 400 and 1200 m, respectively, downstream of a trout farm (see Table 3). None of the field samples in the northern part of the Drammenselva watercourse yielded a positive result when tested against *G. salaris*, neither did the rainbow trout positive control at the trout farm. All extraction blank controls and environmental blank controls were negative, both in qPCR and ddPCR.

### Conventional monitoring methods

At location 1, electrofishing of an area of roughly 300 m^2^ yielded seven juvenile brown trout. Two juvenile brown trout were caught at location 2 after electrofishing an area of *c.*200 m^2^. At location 3, electrofishing was carried out in selected pot-holes along a stretch of 150 m. A high density of brown trout with sizes ranging from juveniles up to 500 g adults was observed. At the fourth location, electrofishing was carried out along a stretch of 200 m. Several minnows *Phoxinus phoxinus* (L., 1758) were observed and many brown trout (juveniles to 300 g) were captured in the stream while electrofishing. No electrofishing was carried out at locations 5, 6 and 7 as none of these locations were suitable for electrofishing. A total of 21 Atlantic salmon with a length of 9.6 cm (± SD 3.6 cm) were caught in Lierelva. The parasite prevalence and intensity on these fish was determined to be 85.7% and 83 parasites (± SD 63), respectively. Throughout the entire electrofishing, no rainbow trout were caught.

## Discussion

In the present study, eDNA monitoring is used for the first time to detect the monogenean parasite *G. salaris* along with two of its hosts, Atlantic salmon and rainbow trout. Detections were successfully obtained both in all singleplex reactions (qPCR and ddPCR) and in a duplex reaction (ddPCR) targeting both *G. salaris* and Atlantic salmon. The prevalence in susceptible Atlantic salmon populations most often reaches 100 % [11]. In general, the infection grows exponentially on non-responding hosts and may reach several thousand individuals per fish [5]. In our study, the *G. salaris* infected Atlantic salmon individuals caught in Lierelva were only moderately infected (prevalence of 85.7%, mean parasite abundance of 83 parasites). Here *G. salaris* eDNA was detected in amounts ranging from 500 to > 350,000 copies per litre of water in the same river stretch. These results strongly indicate that eDNA analysis of samples obtained by water filtering can indeed be used for monitoring the occurrence of *G. salaris* in freshwater ecosystems containing natural Atlantic salmon populations.

Environmental DNA-detection is a promising tool that can be used to supplement or even replace classical surveillance where it produces fast and robust results. This is reflected in the ever growing number of assays being developed to monitor parasites which infect fish. These include both ectoparasites like *Amyloodinium ocellatum* Brown, 1931 [38], *Chilodonella hexasticha* Kiernik, 1909 [39] or *Neobenedenia girellae* Hargis, 1955 [40] and endoparasites such as *Opisthorchis viverrini* Poirier, 1886 [41], *Ichthyophonus* spp*.* [42] and myxozoans [43, 44]. Unlike traditional monitoring, there is no need to kill large numbers of fish or to carry out time-consuming manual examinations. Thus, the eDNA monitoring method has far-reaching potential as it reduces the time and cost of sampling and improves fish welfare. A further advantage of this method is the simultaneous detection of parasite and host. Using the protocol for filtration, DNA-extraction and the analysis we describe here, it is not only possible to detect the parasite *G. salaris* but also two of its hosts within on single sample. With the use of other assays, the presence of virtually any aquatic host-pathogen complex can be detected and monitored, provided that the filter size is appropriate to capture eDNA from the target organism.

The aim of the *G. salaris* qPCR assay designed in the present study was to achieve an optimal combination of both specificity and sensitivity, and the assay was chosen over the one previously published by Collins et al. [32] due to its slightly higher sensitivity. Both the qPCR assay presented in this paper and the qPCR assay designed by Collins et al*.* [32] display a low-level amplification of *Gyrodactylus derjavinoides*. However, this issue was not observed when applying the newly designed primers and probe in ddPCR. Any assay for *Gyrodactylus salaris* targeting the ITS1 region will yield positive results for *G. thymalli* since these two species have nearly identical sequences [33] and it is therefore impossible to differentiate between them in this way. This does not affect the monitoring of *G. salaris* in systems uninhabited by grayling, the host for *G. thymalli*. In systems where grayling occur, negative samples would still indicate the absence of the parasite. A positive detection would certainly require additional examination and attention. Here, one option would be to design assays targeting the more variable mitochondrial cytochrome oxidase gene (see, e.g. Meinilä et al*.* [45], Hansen et al*.* [46]).

In the present eDNA study, as well as for most other applications, the low level of cross-reaction against *G. derjavinoides* when using qPCR poses no problem. If a population of fish were infected with a high number of *G. derjavinoides* and a low number of *G. salaris*, analysis with qPCR could yield ambiguous results. We therefore recommend the use of ddPCR analysis since this method bypasses the problem of cross-amplification. Alternatively, sampling by electrofishing followed by manual examination and standard species identification could be carried out in this particular case.

We detected rainbow trout eDNA at four locations in the northern part of the Drammen watercourse in addition to the sample taken at the trout farm (sample no. 3). We observed an apparent decline in eDNA concentration with increasing distance from the source (area with trout farms, sample nos. 6 and 7). This corresponds with data from studies that examine the dilution effects of eDNA in river ecosystems [47, 48]. However, the number and the distribution of sampling points in this study were not comprehensive enough to examine a gradient thoroughly. Extensive electrofishing at each sampling point produced no evidence for the presence of rainbow trout in the streams. We therefore attribute all positive samples to eDNA discharge/emission from trout farms and assume the areas and streams of the northern part of the Drammenselva watercourse that were tested to be free from wild populations of rainbow trout. The occurrence of these positive samples reveals one of the pitfalls of the eDNA methodology, as it simply points out the presence of eDNA from the targeted organism without verifying the actual presence of the organism within the examined body of water [20, 49, 50]. It does, however, also highlight the sensitivity of this method.

**Fig. 2:**
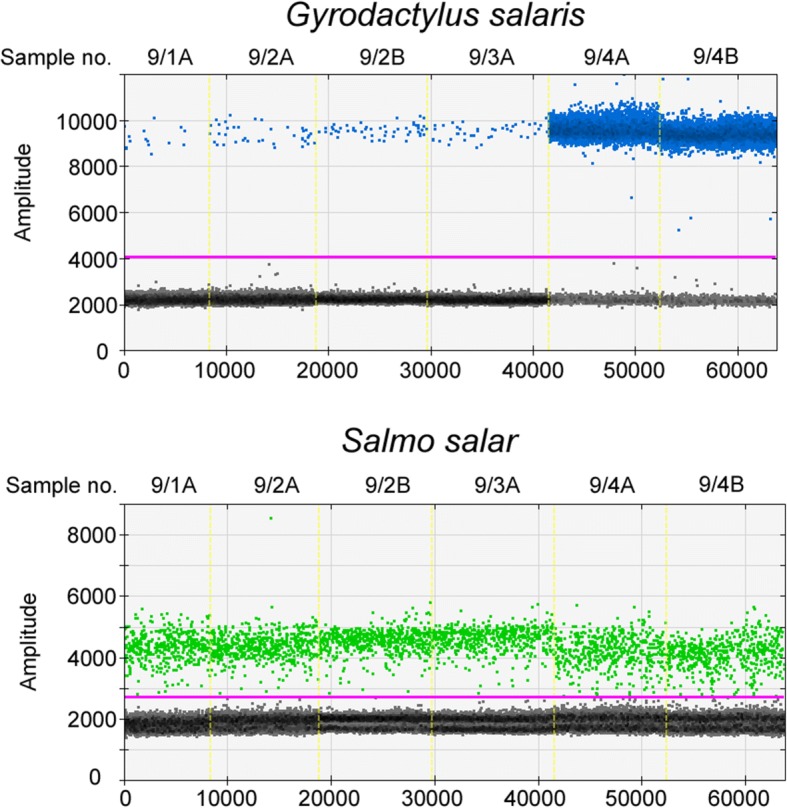
Visual output from the duplex ddPCR for *G. salaris* in Channel 1 (blue) and Atlantic salmon in Channel 2 (green) on the samples taken at Lierelva. Wells containing samples 9/1B and 9/3B are not displayed and were excluded due to insufficient droplet generation. Each blue and green point represents a positive amplification of respective DNA template. The horizontal purple line represents the threshold and the black points represent negative droplets. The eDNA copy number for *G. salaris* is markedly higher in two of the wells containing samples 9/4A and 9/4B. However, the copy number of Atlantic salmon eDNA remains relatively stable in all four samples

One of the four filter samples taken at Lierelva, the river with a known presence of *G. salaris*, displayed a significantly higher signal than the other three filters, even though the very same location was sampled. These results were observed in qPCR, and both the singleplex and multiplex ddPCR reactions. We presume that this is due to one or more whole specimens of *G. salaris* being picked up on this particular filter. The signal difference in qPCR is roughly ten cycles which would suggest a 1000-fold higher amount of eDNA in sample 9/4. This calculation is also reflected in the ddPCR results where an increase from 560 copies/l to 371,440 copies/l was observed. This assumption is substantiated by the fact that Gyrodactylids are reported to consist of roughly 1000 cells [1]. The possibility that one sometimes might catch a whole parasite specimen in the filter does not pose a problem for a simple proof of presence detection, but in fact increases the certainty of the results. However, while some studies have demonstrated a correlation between biomass and eDNA concentration [51], quantification of parasites and establishing an agent-level would, in this case, result in an overestimation of parasite numbers. The use of a pre-filter such as fitting a plankton net in front of the filter with a mesh size small enough to prevent an entire specimen to pass on to the filter may solve this problem of overestimation. In comparison to the results for *G. salaris*, the copy number for Atlantic salmon eDNA was fairly constant in all four samples at an average of 8948 copies (± SD = 945) as displayed in Fig. 2. This indicates a constant emission rate of eDNA into the water by Atlantic salmon which has also been observed in other studies [52].

### Comparison of qPCR/ddPCR monitoring

Quantitative real-time PCR (qPCR) offers the possibility to measure the rate of generation of the amplified product after each cycle, thus making it possible to calculate the amount of copies in the original sample. Previous studies have demonstrated that quantification of biomass and calculation of population size through using qPCR is possible [22, 53]. ddPCR, which now allows the user to operate on a nanolitre rather than on a microlitre scale, enables even more precise detection and absolute quantification of target molecules while simultaneously removing the need for standard curves [51, 54]. Our results demonstrate this precision by detecting both rainbow trout and *G. salaris* at very low copy levels with 22 eDNA copies/l and 560 eDNA copies/l, respectively. Furthermore, this technology has been proven to perform better on inhibition prone samples than the predecessor qPCR [55]. This is a particular advantage when analysing environmental samples which often tend to include PCR inhibitors [56–58]. Our study also shows that ddPCR seems to surpass qPCR regarding specificity, as there was no cross-amplification of *G. derjavinoides* in the *G. salaris* assay although the same primer-probe combinations were used. We speculate that this is due to the lower copy numbers of both target and non-target DNA per reaction (droplets) in the ddPCR system. Ideally, one droplet contains only one copy of the target DNA and only a few non-target copies, thus reducing the possibility of unspecific amplification.

For a more precise monitoring of *G. salaris* and its hosts, further research and development is needed in order to improve the specificity of the *G. salaris* assay to distinguish from *G. thymalli*, as well as to determine when it is no longer possible to obtain a positive eDNA result (limit of detection) when the parasite load per fish drops.

## Conclusions

We have successfully designed and implemented a method for eDNA detection of an aquatic host-parasite system, specifically *G. salaris* and its two hosts Atlantic salmon and rainbow trout. Thus, we demonstrate for the first time that eDNA monitoring can be used for the detection of *G. salaris* and its host Atlantic salmon in natural freshwater systems with a moderately infected salmon population. Furthermore, we have determined the assay we designed to be species-specific and demonstrated the usefulness of eDNA methodology when examining a river system for the presence of *G. salaris*. Within the paper we present a protocol, both field and laboratory, on how to conduct eDNA monitoring of *G. salaris* and Atlantic salmon successfully, using a duplex ddPCR. We show that ddPCR appears to be a better tool than qPCR when screening samples for *G.salaris*. Further studies are needed to determine the limit of detection regarding eDNA and to compare the eDNA signal against fish parasite load in experimental and natural settings.

## Abbrevations

BLASTn: Basic Local Alignment Search Tool
Cq-Value: quantification cycle
CTAB: cetyl trimethyl ammonium bromide
ddPCR: droplet digital PCR
eDNA: environmental DNA
ITS1: internal transcribed spacer 1
MGB: minor groove binder
mtDNA: mitochondrial DNA
NADH: nicotinamide adenine dinucleotide dehydrogenase
qPCR: quantitative PCR
